# Abstracts from Foreign Journals

**Published:** 1901-09

**Authors:** M. P. Ravenel, S. J. J. Harger

**Affiliations:** Philadelphia, Pa.; Philadelphia, Pa.


### ABSTRACTS FROM FOREIGN JOURNALS.

### FRENCH.

UNDER THE DIRECTION OF M. P. RAVENEL, M.D., AND S. J. J. HARGER, V.M.D., OF PHILADELPHIA, PA.

PICRICATED GLYCERIN IN THE TREATMENT OF WOUNDS. This preparation is an oily, citrine colored liquid, consisting of a saturated solution of finely powdered picric acid dissolved in glycerin over a water-bath. Etellin, among others, has employed this solution with much success in canker of the foot, wounds of the foot, as well as of other regions, fistula of the poll and withers, in conjunction with the usual surgical measures, grease, mallenders, and sallenders. It contains the properties of both its ingredients, acting as a local anæsthetic as well as softening the tissues; it stains the tissues yellow.—*Rec. de Med. Vet.*

SHOULDER LAMENESS IN CATTLE FROM SUBSCAPULAR ADENO-TUBERCULOSIS. The subscapular and humeral lymphatic glands may become enlarged from tubercular lesions, and, by pressing upon the branches of the brachial plexus, cause shoulder lameness.

Hamoir has witnessed five such cases. In the first two the lesion was recognized only at the necropsy; in the other three cases a diagnosis of tuberculosis was rapidly made and verified by the post-mortem.—*Annales de Med. Vet.*

BICIPITAL SYNOVITIS. Subject, mare. Suddenly became lame in pasture six months before. Symptoms: Lameness marked, leg stiff and abducted during extension; slight pain on pressure over the point of the shoulder; when the latter was struck with the fist the animal evinced pain by backing quickly. The pain was very acute when the leg was suddenly pulled backward. Raising the opposite leg so as to throw all the weight upon the lame member was very painful. There was no visible alteration of any portion of the leg.

The autopsy revealed a synovitis of the bursa of the biceps muscle, ulceration of the cartilage of the bicipital groove, and osteitis of the humerus; the shoulder-joint was intact.

The lameness from this lesion could, on account of the abduction and difficulty of flexion, be confounded with other forms of shoulder lameness. In the absence of any deformity of the region, lameness at this point may be overlooked. The diagnosis under such condition depends: 1. The pain during support when the opposite member is raised. 2. Difficulty of advancing the leg and want of flexion of the forearm. 3. The acute pain when the leg is suddenly drawn backward so as to tense the biceps muscle.—*Detroye, Rec. de Med. Vet.*

Orthonitrophenylpropionic Acid for Detecting Sugar in the Urine. This acid (nitropiol) is prepared by German chemists in the form of tablets. One is dissolved in 10 c.c. of water, and a few drops of urine are added; after boiling the solution turns blue if glucose is present. This coloration is due to the reduction of the nitropiol, which is decomposed into indigo blue. This reaction is more delicate than Fehling's solution, and is not affected by uric acid or creatinin.—*Revue Pharmaceut.*

Chirol. This is a solution of certain resins and fatty oils in alcohol and ether. It is employed by surgeons to protect the hands from infection. The latter are disinfected and then plunged for a few seconds into the chirol; when dried it forms a tough, elastic coating. It can be removed by washing the hands in alcohol and applying cold cream.—*Revue Pharmaceut.*

Pleurodynia in the Horse. Among the remounts of the Bavarian Army, of which 1000, from three to four years old, are purchased annually, Army Veterinarian Sigl has found many cases of pleurodynia among those which pass through dealers' hands and have to be transported by railroad for long distances. As causes he recognizes fatigue, currents of air, enforced standing position in railroad cars, and other unfavorable conditions incident to railroad transportation.

The symptoms are: Stiffness in gait, rigid back and loins, difficulty in backing and turning, extension of head and neck, sunken eyes, retracted flank, painful expiration, cough absent, pulse 50 to 60, temperature from normal to 40° C., auscultation and percussion which are painful, normal unless there is pleuritic inflammation, and loss of appetite. In mild, uncomplicated cases recovery follows in from four to six days.

The prognosis is benign, excepting in cases complicated with pleurisy. According to its gravity the author recognizes three degrees of pleurodynia in the horse:

1. Rheumatismal myositis.
2. Rheumatismal myositis complicated with dry, bilateral pleurisy.
3. Rheumatismal myositis with pleurisy, at first dry and afterward exudative.

The treatment consists in quiet surroundings, salicylate of sodium, sulphate of sodium, and, if necessary, alcohol and rectal clysters.—*Annales de Med. Vet.*

Treatment of Indolent and Summer Wounds. Dr. Ducasse treats summer wounds, so difficult to heal, with tincture of iodine. The skin around the ulcer is shaved and the ulcer is then painted with the iodine morning and night, leaving the parts uncovered. In four or five days a dry scab forms, and cicatrization gradually follows. Laquerière, while sojourning in Algeria, employed almost exclusively iodine in the treatment of harness wounds with the best results. Iodine is an antiseptic and causes hypernutrition.—*Annales de Med. Vet.*

The Action of Solar and Diffuse Light upon the Tubercle Bacillus in Sputum. Koch and others have experimentally demonstrated that cultures of this bacillus when exposed for an undetermined period to solar or diffused light are attenuated or destroyed.

Jousset has inoculated guinea-pigs with sputum exposed to such light for periods of different duration. Some of them died from tuberculosis in from nineteen to thirty-two days; some showed the bacillus only in the ulcer and the abscess at the seat of inoculation; others survived, and were entirely free from bacilli.

The author concludes: In a certain number of cases the tuberculous sputum is completely sterilized by light; it is always strongly attenuated, since the apparent health of inoculated animals is preserved, the weight increases; the bacillus is found only at the seat of inoculation, and a large number survive. The attenuation varies with the duration of the exposure.

These phenomena insure a certain amount of protection against the pathogenesis of sputum deposited on the highways and other public places.—*Annales de Med. Vet.*

Experimental Vaccination against Canine Distemper. Ligonières has recently described the microbe of distemper in the dog. It is a long bacillus which grows in bouillon of peptone, without cloudiness, and forms small granulations which fall to the bottom. This bacillus has been isolated from the blood and organs of diseased dogs sacrificed before the period of secondary infection. Cultivated in peptone bouillon, the microbe becomes gradually attenuated with the age of the culture, and by reinoculating ordinary bouillon at the end of variable periods cultures of diverse attenuations can be obtained; such cultures, by passing again through a dog, assume their primitive virulence.

An attenuated culture inoculated under the skin on the inside of the thigh produces a painful swelling, which commences to disappear at the end of forty-eight hours without any general symptoms. If the culture is more virulent œdema and sometimes an abscess, elevation of the temperature, and recovery follow. With such cultures Phisalix has made vaccinations against distemper. At first a very attenuated culture was used ; these were followed by three or four cultures of greater virulence.

Dogs thus treated were immune against distemper after intravenous injections of the virulent culture or by cohabitation with infected animals ; some of them were in contact with diseased animals for a long time ; in others the infected mucous discharges were smeared around the nostrils. There was no infection.—*Rec. de Med. Vet.*

CAUSE AND TREATMENT OF PERIODIC OPHTHALMIA. During an outbreak of this disease among 150 horses in the garrison of Auxonne, Dor succeeded in isolating the specific agent of this disease, and reproduced the latter experimentally in the horse.

This microbe, found in the ocular media, is a staphylococcus having some resemblances with the staphylococcus pyogenes doris, and does not develop in alkaline media. The periodicity of the disease is explained thus : The media of the eye are acid, a favorite condition for the growth of the microbe ; during the progress of an attack of the disease the media becomes alkaline, the growth of the microbe becomes arrested, and the eye clears up. When the alkalinity disappears the remaining microbes again become active, and a second attack occurs.

*Experimental Reproduction of the Disease.* Inoculation of four drops of a virulent culture diluted five times into the vitreous humor produces an attack of ophthalmia of mean intensity with a hypopion continuing from twenty to twenty-five days, and recurring in from twenty-five to thirty-nine days. From these experimental reproductions the author maintains the specificity of the microbe.

*Therapeutics.* Iridectomy and instillations of atropia have given favorable results, but are not very practicable.

Basing himself upon the low vitality of the microbe in an alkaline medium, Dor endeavored to alkalinize the ocular media by intravenous injections of caustic soda, carbonate of soda, and carbonate of lithia, but without any success.—*Journ. de Med. Vet. et de Zootech.*

Transmission of Sarcoptes Minor from the Cat to Bovidæ. Chapellier relates an instance of a cat suffering with mange having been kept in the cow stable and infecting three cows. Sometime after the beginning of the cohabitation the cows showed the first signs of the disease, which was accompanied by itching, licking of the parts, falling out of the hair, and small papules covered with reddish scabs over larger or smaller patches over the neck, back, loins, and croup. The owner had seen the cat sitting on the backs of these animals, and hence the opinion that the cat was a causative factor. In the epidermic débris around the base of the hair of the cat the *sarcoptes minor* was found.

The examination of the scabs from the skin of the cows proved negative. The animals recovered in about eight days with the application of simple remedies. According to Poilliet, mange is transmissible from the cat to cattle.—*Rec. de Med. Vet.*

Etiology of Infectious Enteritis and Bronchopneumonia in Calves. Every veterinarian and farmer is familiar with the serious loses entailed by infectious diarrhœa in calves. It manifests itself within the first six or eight weeks, sometimes immediately after birth. Moussu and Nocard have recently demonstrated that infectious diarrhœa, infectious bronchopneumonia, as well as infectious arthritis (arthritis of young animals), in calves are so many diverse manifestations of the same disease which sometimes sweeps entire communities. The bronchopneumonia usually follows an amelioration of the enteric trouble, and follows a much less rapid course, the animal seldom dying before the eighth week. Nocard recently investigated this disease in Ireland at the request of the Department of Agriculture, and seems to have conclusively demonstrated its cause. "White scours" was at this time very prevalent, and in most cases complicated with "lung disease," catarrhal pneumonia, of variable degree, and sometimes with fibrinous arthritis.

Nocard made a bacteriological examination of the contents of the femoro-tibial articulation affected with arthritis and obtained from a calf suffering from a subacute form of infectious enteritis. A variety of microörganisms were found, and among them was a small immobile bacterium, not staining with the method of Gram, not coagulating milk, nor growing upon potato, belonging to a group of organisms called "Pasteurella," the group of hemorrhagic septicæmic bacteria, and possessed of extreme pathogenic properties.

Cultures of this bacterium were made. A few drops injected into the veins of the rabbit or the peritoneum of the guinea-pig was fatal in six to eight hours. The post-mortem showed intense lesions of hemorrhagic septicæmia corresponding with those of infectious enteritis. The microbe existed in the blood, all the viscera, and in the umbilical cord.

Artificial reproduction of the disease was successful. A calf brought from a community where the disease was not known to exist was inoculated, April 16th, with a culture of the bacterium, and the following day died—a very acute case, showing the usual symptoms and post-mortem lesions. From the blood, pleural and pericardial effusion, spleen, liver, and mesenteric ganglia of these animals colonies of the microbe were reproduced. This experiment was repeated twice with identical results. The second calf was obtained from a farm infected with "white scours." It was about four weeks old and had recovered from an attack of the disease. The inoculation was negative, which seemed to prove that a first attack confers at least a temporary immunity.

The probable mode of infection is through the umbilicus, where the microbe is always found at the time that the fœtus passes through the vagina, which always contains numerous microbes, or by contact with the soiled litter on the stable floor.

The prophylactic treatment consists in plenty of clean bedding, washing out the vagina, as well as the buttock of cow with an antiseptic solution just before calving; tying and cutting off the umbilicus, washing it with an antiseptic solution, and painting it with collodion.

A Gastro-intestinal Affection in the Dog and the Cat. Ben-Danou has observed a peculiar affection of this nature in a large number of dogs and cats always presenting the same phenomena, at times accompanied by cerebral and spinal symptoms and a high mortality.

*Symptoms.* Profound adynamia, arching of the back, retraction of the abdomen, dryness of the mouth, fetid breath, excessive thirst, vomiting, excrements "formed" and yellow; there is neither diarrhœa nor constipation; apyrexia, three or four days before death, the temperature being subnormal; sometimes there is gangrenous stomatitis; salivation, gangrene of the tongue, and perforation of the cheek; the subject dies in coma or in epileptic convulsions.

The necroscopical lesions in the mouth, stomach, and intestines are of an inflammatory nature, but their acuteness is not in accord with the gravity of the symptoms. The phenomena are most probably due to an autointoxication of gastro-intestinal origin.

This case once more calls attention to the frequent but unstudied autointoxications witnessed in animals kept under improper dietetic conditions. The writer has seen in the dog fatal instances apparently identical with the conditions described above.—*Rec. de Med. Vet.*

POSITION OF THE COW IN REDUCING EVERSION OF THE UTERUS. Monsarrat's procedure is the following: The hind quarters, whether the cow stands or lies down, are maintained in the upright attitude by means of a rope passed around the base of the tail (phlebotomy knot) and fastened to the ceiling. If the cow is standing she is made to lie on the sternum by flexing the canon upon the forearm with a rope or quiler strap. The forward slant of the axis of the body assists the uterus to gravitate into its position. No serious effects have been seen from tying the rope around the tail.—*Rec. de Med. Vet.*

SEAT OF ABSCESSES AND FISTULÆ IN ARTHRITIS OF THE FOOT. Suppurative arthritis of the foot is followed by articular abscess and fistula, which appear at certain contact points, due to the anatomical disposition of the coronary region. The fistulæ always appear at the level of the lateral ligament, and have their origin in the necrosis of these ligaments which, on account of their low degree of nutrition, cannot react against the inflammatory agents, become the seat of necrosis that slowly perforates the skin and produces a fistula at that level.—*Rec. de Med. Vet.*

ETIOLOGICAL AGENT OF VACCINE AND VARIOLA. Dr. Funck, of the University of Brussels, experimented to prove that variola is not a microbic disease. These, when present, result from accidental contamination. After three months they disappear from the vaccine, which is still capable of producing the characteristic pustules. The causative agent, according to the author, is a protozoon which he calls the sporidium vaccinale. It can be distinguished with a microscope of low power, and may exist in three different stages, according to its degree of development. It has been found in all vaccines examined.

The inoculation of a culture or a sterile emulsion of the protozoon reproduced the symptoms of the disease. In the calf inoculated with the spores mixed with a drop of bouillon, the characteristic lesions of the pustules appear toward the sixth day. Calves inoculated with a fresh emulsion of the protozoon were immune to subsequent inoculations with vaccine which will not produce the least reaction. This parasite is always present in the pustules produced by the successive inoculations of three or four calves. It is found in both the variola and the vaccine pustules. The etiology of vaccine and of variola is therefore identical.

During its complete evolution this protozoon assumes three different forms :

1. It is round, of a bright green reflection, 2 to 10$\mu$ in diameter, and when heated to 37° C. shows characteristic movements.

2. More or less elongated ovoid cells with a nucleus toward the periphery, whose protoplasm encloses small, round, bright green bodies analogous to the preceding, and measuring 1 to 3$\mu$ in diameter. These are the epidermic cells containing the protozoon.

3. Finally, a form very frequently seen in all vaccines and consisting of a mulberry-like mass (morula), sometimes round with or without a double contour and measuring 25$\mu$ ; sometimes oval and measuring 30 to 35$\mu$ in length and 20 to 25$\mu$ in width. These are the sporoblasts filled with spores.—*Annales de Méd. Vét.*

---

*Offers Himself as a Tuberculosis Subject.* In view of the interest taken in the question of whether or not animal tuberculosis can be communicated to human beings, T. L. Monson, State Dairy Commissioner of Colorado, offers himself as a subject for thorough test, provided a suitable annuity for his family shall be assured in case of fatal results.

---

The Trunk Line Association and the Southern Passenger Association have already announced a one and one-third rate fare, certificate plan, to the A. V. M. A. meeting.

